# 10-HDA, A Major Fatty Acid of Royal Jelly, Exhibits pH Dependent Growth-Inhibitory Activity Against Different Strains of *Paenibacillus larvae*

**DOI:** 10.3390/molecules23123236

**Published:** 2018-12-07

**Authors:** Mária Šedivá, Maroš Laho, Lenka Kohútová, Andrea Mojžišová, Juraj Majtán, Jaroslav Klaudiny

**Affiliations:** 1 Institute of Chemistry, Slovak Academy of Sciences, Dúbravská cesta 9, 845 38 Bratislava, Slovakia; maria.sediva@savba.sk (M.Š.); maros.laho@savba.sk (M.L.); lenka.kohutova@savba.sk (L.K.); 2 Institute of Molecular Biology, Slovak Academy of Sciences, Dúbravská cesta 21, 845 51 Bratislava, Slovakia; juraj.majtan@savba.sk; 3 Veterinary and Food Institute in Dolny Kubin, Janoškova 58, 02601 Dolný Kubín, Slovakia; andrea.mojzisova@svpu.sk

**Keywords:** honeybee, 10-hydroxy-2-decenoic acid, antibacterial, bacterial spores, royal jelly, worker jelly, larval jelly, ERIC-PCR genotyping, American foulbrood

## Abstract

*Paenibacillus larvae* (*P. larvae*) is a bacterial pathogen causing American foulbrood (AFB), the most serious disease of honeybee larvae. The food of young larvae could play an important role in the resistance of larvae against AFB. It contains antibacterial substances produced by honeybees that may inhibit the propagation of the pathogen in larval midguts. In this study, we identified and investigated the antibacterial effects of one of these substances, *trans*-10-hydroxy-2-decenoic acid (10-HDA), against *P. larvae* strains including all Enterobacterial Repetitive Intergenic Consensus (ERIC) genotypes. Its inhibitory activities were studied by determining the minimum inhibitory concentrations (MICs). It was found that 10-HDA efficacy increases substantially with decreasing pH; up to 12-fold differences in efficacy were observed between pH = 5.5 and pH = 7.2. *P. larvae* strains showed different susceptibility to 10-HDA; up to 2.97-fold differences existed among various strains with environmentally important ERIC I and ERIC II genotypes. Germinating spores of the pathogen were generally more susceptible to 10-HDA than vegetative cells. Our findings suggest that 10-HDA could play significant role in conferring antipathogenic activity to larval food in the midguts of young larvae and contribute to the resistance of individual larvae to *P. larvae*.

## 1. Introduction

American foulbrood (AFB) is a redoubtable infectious disease of honeybee (*Apis mellifera*) colonies. It is the most destructive brood disease that causes financial losses to beekeepers and farmers worldwide. The causative agent of AFB are spores of the Gram-positive bacterium *Paenibacillus larvae* that may contaminate larval food. The infectivity of larvae depends on their age (the most susceptible are larvae younger than 36 h) and on the dose of spores in food [1,2]. A precondition for a fatal ailment of larvae seems to be the massive proliferation of vegetative bacterial cells in the larval midgut [3], associated with the production of enzymes and substances that help the bacteria to penetrate through the peritrophic matrix and gut epithelium into the hemocoel [4,5,6]. The subsequent cell proliferation in hemocoel causes the death of the larva, its decomposition and creation of billions of spores that under suitable conditions drive intra-colonial and inter-colonial larval infections that, without the beekeeper’s intervention, can lead to the collapse of the colony [2,7,8].

It has been found that speed of the progression and the severity of AFB in colonies depend on the virulence of *P. larvae* strains. Strains with the ERIC (Enterobacterial Repetitive Intergenic Consensus) I genotype are less virulent to larvae and kill them mostly after cell capping. The strains possessing the ERIC II, ERIC III and ERIC IV genotypes are highly virulent and kill larvae before cell capping [9,10]. The highly virulent strains paradoxically cause the slower destruction of colonies than less virulent ones. This is associated with the ability of honeybees to detect and remove diseased larvae from the hive (hygienic behaviour), which is more effective when larvae die before cell capping [11,12].

The control of AFB is based on prevention, early detection, disinfection and treatment strategies in the case of infection and the destruction of clinically infected hives by burning [2,8,13,14,15,16]. The prospective way to fight against the disease seems to be the breeding of honeybee lines that are more resistant to AFB. Such lines should possess more effective individual and social defense mechanisms that act in colonies against AFB [2,11,17,18]. It has been shown that the selective breeding of colonies based on the hygienic behavior of bees leads to increased AFB resistance of colonies [15,19,20].

We assume that one possible but poorly explored social protective mechanism of larvae and colonies against AFB could be based on the antimicrobial substances present in larval food and their ability to prevent massive proliferation of bacterial cells in the midguts of individual larvae. It is known that the food of larvae younger than 3 days, i.e., larval jelly (LJ) including royal, worker and drone jelly (RJ, WJ and DJ) [21], contain antimicrobial substances such as peptides royalisin/defensin1 and jelleines [22,23,24], the protein apalbumin2a [25] and some hydroxy or dicarboxylic derivatives of medium-chain fatty acids including *trans*-10-hydroxy-2-decenoic acid (10-HDA) [26,27,28,29] that produce nurse honeybees. At present, there is a little information about in vitro effects of these substances against *P. larvae* and no information about their *in vivo* action in larvae. There are several reports demonstrating the antibacterial effects of WJ [30], RJ [31] and their extracts [32] on *P. larvae* and a few demonstrating the inhibitory effects of native defensin1 [33,34], recombinant defensin 1 [35] and protein apalbumin 2a [25] on the growth of the pathogen.

*Trans*-10-hydroxy-2-decenoic acid or (*E*)-10-hydroxydec-2-enoic acid (IUPAC name) (10-HDA) is the most abundant fatty acid (FA) and a major lipid component of RJ [26,36]. This unique FA shows various biological and pharmacological activities [37,38,39] and is used as a marker of RJ quality and authenticity [40]. Although several studies have demonstrated its antimicrobial activity against various Gram-positive and Gram-negative bacteria (mostly human pathogens) [26,27,28,41], no study has examined its antibacterial properties against *P. larvae*.

Therefore, we aimed to investigate the antibacterial effect of 10-HDA against *P. larvae* using reference strains and field isolates having all known ERIC genotypes. Due to the fact that the effect of 10-HDA in honeybee larvae could be affected by different levels of acidity of consumed LJ and by the pH value of the midgut environment, its antibacterial efficacy was studied in media with different pH levels. Furthermore, the susceptibility of vegetative cells and germinating spores of *P. larvae* to 10-HDA was compared. Based on the obtained findings, we assessed the potential of 10-HDA to inhibit the propagation of *P. larvae* in larval midguts, either individually or with the help of other factors associated with LJ. 

## 2. Results

### 2.1. ERIC Genotypes of Bacterial Strains

ERIC genotypes were determined for all *P. larvae* strains used in this work. Representative results from genotyping of selected strains are shown in supplementary materials in Figure S1. The individual strains and their genotypes are given in Table 1. The characterization confirmed previously specified ERIC I genotypes of reference strain CCUG 28515 and LMG 9820 (identical with DSM 7030) [10]. Another three reference strains, i.e., CCM 4483, CCM 4486 and CCM 39 had the ERIC I, ERIC II and ERIC III genotype, respectively. The reference strain CCM 38 contained two morphologically different strains, one with ERIC III and the other with the ERIC IV genotype. All field isolates of *P. larvae* had the same ERIC II genotype.

### 2.2. Effect of pH and Substance PIPES on Growth of P. larvae

**Figure 1:**
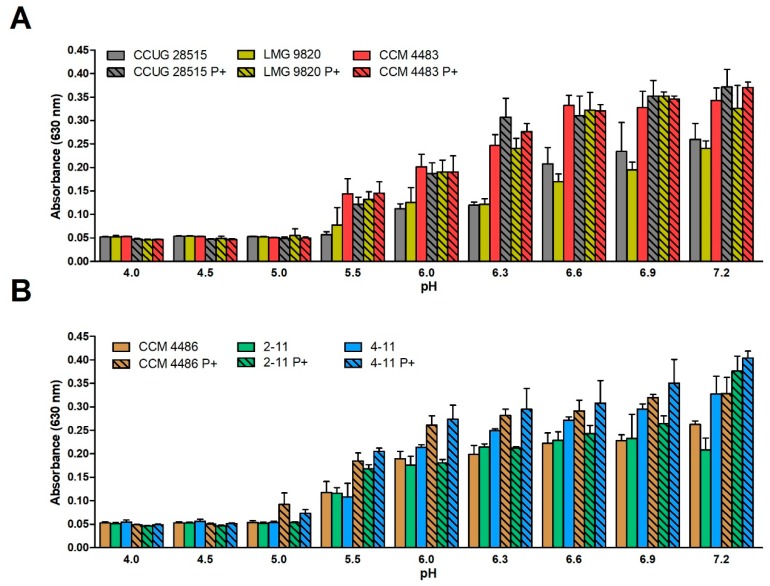
Growth of selected *Paenibacillus larvae* strains representing two environmentally significant Enterobacterial Repetitive Intergenic Consensus (ERIC) genotypes at various pH in MYPGP media. (**A**) ERIC I genotype, (**B**) ERIC II genotype. P+ indicates the growth of bacteria in MYPGP+P medium containing the buffering compound PIPES. Data are expressed as mean values with standard deviations.

Before studying the inhibitory effect of 10-HDA against *P. larvae*, we examined suitable conditions for bacterial cultivation. Firstly, we started to cultivate the bacterium in MYPGP medium which is most frequently used for this purpose. The preliminary tests showed that adding 10-HDA (weak acid) to the medium having low buffering capacity caused the not negligible reduction in the pH of the medium dependent on FA concentration. Therefore, MYPGP medium was adjusted with 50 mM PIPES to increase its buffering capacity and the modified medium was designated as MYPGP+P medium. Then, we examined the effect of pH on the growth of six selected strains of *P. larvae* carrying two environmentally significant ERIC genotypes in both media, MYPGP and MYPGP+P, to determine the effect of PIPES on bacterial growth. The examined pH range included pH values at which this bacterium can occur in its natural environment, beginning from pH = 4 in LJ to pH = 7.2 used ordinarily for laboratory cultivations. The results of these tests are presented in Figure 1.

It was found that the bacterial strains did not grow at pH = 4 or pH = 4.5, and the most of them did not grow at pH = 5. They started to grow at pH = 5.5 and the level of growth rose with increasing pH to pH = 6.9–7.2. There were no substantial growth differences between the strains possessing the ERIC I and ERIC II genotypes. Individual strains showed partial differences in the bacterial mass grown up during the cultivation. The presence of 50 mM PIPES in the medium had a generally positive effect on the bacterial growth of all strains.

### 2.3. Efficacy of 10-HDA Against P. larvae at Various pH Levels

**Figure 2:**
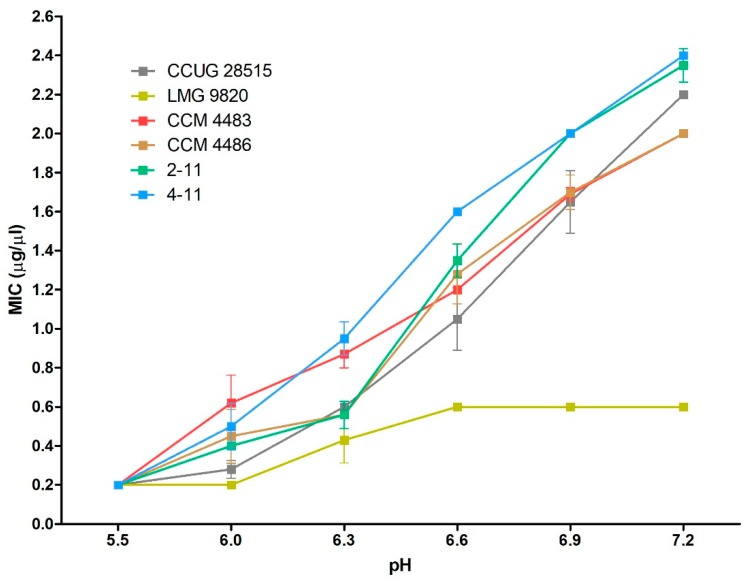
Effect of pH on the antibacterial efficacy of 10-HDA in selected *P. larvae* strains. Minimum inhibitory concentration (MIC) values are graphically depicted to compare pH-dependent susceptibility profiles of individual strains to 10-HDA. Most strains of the ERIC I and ERIC II genotypes showed similar profiles of pH-dependent susceptibility to 10-HDA. Data are expressed as mean values with standard deviations.

The efficacy of 10-HDA against vegetative cells of six bacterial strains (used previously in the growth tests) was estimated by the determination of minimum inhibitory concentrations (MICs) in media with the pH between 5.5 and 7.2. The results are shown in Figure 2. 10-HDA had the strongest inhibitory effect on the growth of *P. larvae* at pH = 5.5. The inhibition at this pH occurred at the same concentration of 0.2 μg/μL of 10-HDA in all tested strains. With increasing pH, the susceptibility of *P. larvae* to FA continuously decreased in five strains and reached MIC values greater than 2 μg/μL at pH = 7.2. The strains with the ERIC I and ERIC II genotypes showed a similar dependence of susceptibility on pH, except for one strain with the ERIC I genotype, LMG 9820. This strain did not show pH-dependent susceptibility between pH = 5.5–6 and 6.6–7.2. We assume that this could be connected with the high susceptibility of this strain to 10-HDA.

### 2.4. Susceptibility of Culture Strains, Field Isolates, Vegetative Cells and Spores of P. larvae to 10-HDA

**Figure 3:**
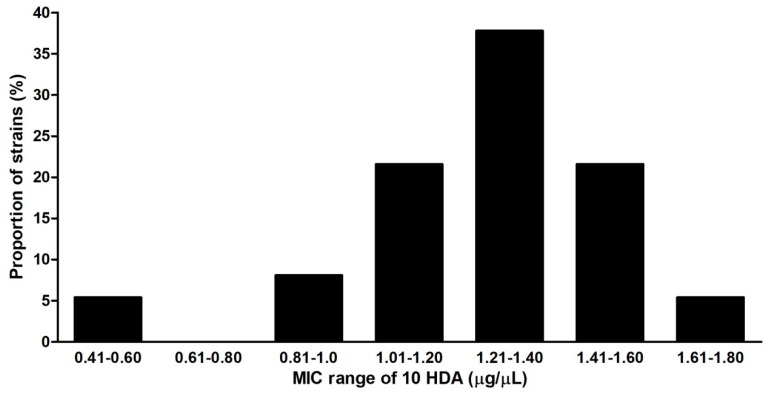
Frequency of *P. larvae* strains having different MICs in a group of strains with ERIC I and ERIC II genotypes.

The susceptibility of seven reference strains and 33 Slovak field isolates to 10-HDA was compared by the determination of MICs at pH = 6.6 (assumed to be the highest pH that may occur in the midguts of young larvae). The determined MIC values ranged between 0.6 and 2.8 μg/μL (Table 1). The strains with ERIC I and ERIC II genotypes were more susceptible to 10-HDA (MICs between 0.6–1.2 and 0.6–1.78 μg/μL, respectively) than those with ERIC III and IV genotypes (MICs 2.6 and 2.8 μg/μL). The results showed that there was relatively high variation in the susceptibility of *P. larvae* strains to 10-HDA considering all ERIC genotypes. The maximal difference in MIC values was 4.67-fold. The strains with ERIC I and ERIC II genotypes showed less variation in susceptibility as the maximal MIC difference was 2.97-fold. Among the tested strains, distinct MICs occurred in various frequencies. The most representative strains were those having MICs between 1.21 and 1.4 μg/μL (Figure 3). The susceptibility of vegetative cells and germinating spores to 10-HDA were compared with strains used in the previous tests. The germinating spores of most strains had MIC values of 10-HDA lower than vegetative cells (Table 2). This result suggests that germinating spores are generally more susceptible to 10-HDA than vegetative cells.

## 3. Discussion

Honeybee colonies differ in their resistance to AFB, in terms of their ability to tolerate certain levels of contamination in bees and food stores with *P. larvae* spores without clinical signs of disease [2,42,43]. Several mechanisms are assumed to act in larvae and colonies influencing the resistance of colonies against AFB. These mechanisms are: (1) The ability of nurse bees to remove spores from contaminated honey by honey-stopper [44,45], (2) changes in the midgut peritrophic membrane with increasing age of the larva connected with the lower ability of the pathogen to penetrate into the hemocoel [46,47], (3) the presence of some microbial species in the larval midgut that inhibit the propagation of *P. larvae* [48,49,50], (4) pathogen-induced immune responses including the expression of antimicrobial peptides [51,52,53] and other immune factors [54] in larvae, (5) the presence of unknown non-induced substance(s) in 2- to 4-day-old larvae inhibiting the growth of *P. larvae* [32,55], (6) the hygienic behavior of honeybees consisting in the detection and removal of diseased larvae from a colony before the pathogen reached the spore stage [11,15,19,56]. 

Besides these mechanisms, several studies have suggested that resistance of colonies to AFB could also be associated with larval food. Rose and Briggs [30] demonstrated that WJ collected from an AFB resistant line was more effective in inhibiting spore germination and in reducing the numbers of vegetative cells of *P. larvae* than the WJ from a susceptible line. Hornitzky [31] observed that RJ has bactericidal effect on vegetative cells of the pathogen within 5 min. The authors of these two studies suggested that the antibacterial effects could be mediated by 10-HDA which at the time was the only known antibacterial substance in RJ. Crailsheim and Riessberger-Gallé [32] found that water-ethanol extracts of RJ and WJ inhibited the in vitro growth of *P. larvae*; the RJ extract showed greater effectiveness than WJ extracts. Furthermore, it was revealed that the LJ peptide defensin1 has in vitro inhibitory activity against *P. larvae* [33,34,35]. It was documented that the content of the peptide varies in RJ and WJ samples collected from different colonies as well as in RJ samples collected in one colony [57]. Moreover, Bíliková et al. [25] identified the protein apalbumin2a in RJ, which also inhibited the growth of *P. larvae in vitro*. These findings suggested that defensin1 and apalbumin2a could be additional substances contributing to the antipathogenic activity of larval food and AFB resistance.

In the present work, we showed for the first time that 10-HDA inhibits the growth of vegetative cells and germinated spores of *P. larvae.* We demonstrated that the antibacterial efficacy of the 10-HDA depends on pH and the susceptibility of individual strains of the pathogen to FA. The strains possessing ERIC I and ERIC II genotypes causing AFB [8,58] seem to have similar susceptibilities to FA. The strains with ERIC III and IV genotypes isolated from colonies in the past and currently available only from culture collections were less susceptible to FA than the ERIC I and ERIC II strains. The molecular reasons for these differences among distinct ERIC genotypes are unknown.

The growth of *P. larvae* and the efficacy of 10-HDA against the different strains of the bacterium were examined here under conditions that partially resemble those occurring during the development of young larvae. The bacterial incubations were done at 35 °C, which is the common temperature in hives and at pH levels assumed to occur in larval food and the midguts of young larvae (pH = 4–6.6). This enabled us to make some conclusions regarding the action of *P. larvae* in the natural honeybee system. We did not observe the growth of *P. larvae* in MYPGP media at pH = 4 or pH = 4.5, and some strains did not grow at pH = 5.0. This suggests that pH itself has bacteriostatic and/or bactericidal effect on the vegetative cells of the bacterium. A lethal effect of saline solution at pH = 4 on vegetative cells within 20 min has been described by Hornitzky [31]. However, our results suggest that the pH range with a detrimental effect on bacteria is higher than pH = 4.

The efficacy of 10-HDA against most of the tested bacterial strains was substantially affected by the pH. The inhibitory potency of FA increased when the pH decreased from 7.2 to 5.5. This suggests that the un-ionised molecules of FA are more antibacterial effective than ionised molecules. This phenomenon seems to be typical for medium-chain FAs because similar pH-dependent potency against some bacteria was observed at caprylic (C8:0), capric (C10:0) and lauric (C12:0) acids [59,60]. The fact that pH has a strong effect on the inhibitory activity of 10-HDA is of great importance due to its possible impact on the ability of this FA to inhibit the growth of pathogen cells in the midguts of young larvae. It is likely that the midgut environment of larvae can have variable pH. The source of pH variations could be genetic factors, the age of the larvae and differences in the acidity of larval food consumed by individual larvae. Chauvin [61] reported that the larval midgut is at pH = 6.8, Bailey and Ball [1] mentioned that the pH in larval intestine is about 6.6 and Wardell [62] stated that the pH is 5–5.5. It seems that the pH in the midgut of young larvae consuming RJ or WJ may have lower values and shows larger variations in values than in older larvae. The reason is that the LJ may show considerable differences in pH and acidity; in RJ, the pH can vary between 3.4 and 4.5 and the acidity (volume of 0.1 N NaOH in mL needed for the adjustment of 1 g of jelly to pH = 7.0) can be between 3.0 and 6.0 [40]. Thus, it is possible consider that 10-HDA at the same concentrations will have greater inhibitory activity in the midguts of young larvae consuming more acidic jellies than in those consuming weakly acidic ones. Differences in inhibitory activities could be as much as 3–8 times between pH = 5.5 and 6.6 according to our results.

Current knowledge about *P. larvae* pathogenesis [3,6,8] suggests that the inhibition of bacterial growth in the larval midgut could forestall the invasion of bacterial cells into larval hemocoel and enable individual larvae to survive the infection. The determination of the MICs of 10-HDA for 37 epidemiologically significant *P. larvae* strains having ERIC I and ERIC II genotypes allowed us to think about the possible antipathogenic action of this FA in midguts of individual larvae. The determined MICs (0.6–1.78 μg/μL) can be considered as concentrations of 10-HDA molecules that should inhibit the multiplication of vegetative cells of the pathogen in the midguts of young larvae at pH = 6.6 (pH used for the MIC determination). The question is whether 10-HDA reaches such inhibitory concentrations in the larval midguts. The contents of 10-HDA have been analyzed in numerous RJ samples of various geographic and hive origins and were found to vary between 0.75% and 6.4% [63,64,65,66,67,68,69,70,71,72]. These percentages correspond to 7.5–64 μg of 10-HDA per 1 mg of RJ. From these data, it can be deduced that RJ containing such amounts of 10-HDA could be diluted from 4.2 to 107 times in the larval midguts in order to exert the MIC within ranges 0.6 and 1.78 μg/μL. The given dilution data (its high values) indicate that the MICs may be theoretically reached in larvae consuming food with low amounts of 10-HDA in cases when infections are induced by more susceptible *P. larvae* strains. Nevertheless, we suppose that the real antipathogenic effect of 10-HDA in the midguts of larvae may be much stronger than indicated dilution data. Several factors can have a positive effect on the inhibitory activities of FA in larvae. Firstly, the inhibitory effect can be multiplied at lower pH assumed to occur in the midguts of young larvae (documented here). Secondly, the inhibitory effect is generally higher against germinating spores (the primary event at pathogen’s infection) than against vegetative cells (also documented here). Thirdly, the inhibitory activity of 10-HDA may be modified by the inhibitory activity of additional proteinous and lipidic antibacterial substances of LJ [33,34,69]. It is likely that the combined action of more inhibitory substances will exhibit much stronger antipathogenic activity in larval midguts than 10-HDA itself. Fourthly, we suppose that the antipathogenic activity of LJ is modified by digestion in larval midguts. We believe that the hydrolytic processes acting during digestion increase the concentrations of free medium-chain FA molecules in the midgut in comparison with their concentrations present in LJ. The free molecules can arise by the hydrolysis of FA monoesters and diesters which together bind molecules of 10-HDA and other abundant medium-chain FAs. The presence of such esters in RJ was revealed by Noda et al. [73]. Thus, the inhibitory activity of larval food processed in midguts might be higher than the inhibitory activity of the consumed natural LJ. The observations of Crailsheim and Riessberger-Gallé [32] support this scenario. They found that the water-ethanol extracts of 2–4 days old larvae were more active than extracts prepared from RJ and WJ samples obtained from the same colonies (extracted LJ samples had the same weights as the larvae). They explained this by the presence of unknown antibacterial substance(s) produced by the larvae. Our scenario represents a possible other explanation or, at least, suggests that hydrolytic processing of the larval foods in extracted larvae contributed to the higher activity of larval extracts. Besides these factors with positive effects on the inhibitory activity of 10-HDA in larval midguts, it is necessary to mention that WJ generally contain lower portions of lipid fraction [74] containing FAs and show lower activities against *P. larvae* than RJ [32]. However, at present there is little information about variations of 10-HDA contents in WJs within a colony and among colonies [63,75]. Taken together, these findings suggest that 10-HDA participates in determining the anti-*P. larvae* activity of larval food in the midguts of young larvae. The final inhibitory effects of 10-HDA in individual larvae might depend on amounts of free molecules of 10-HDA occurring during digestion of RJ and WJ in the midgut, on the effects of other factors supporting the inhibitory activity of 10-HDA (pH and the action of other antibacterial substances of LJ) and finally also on the susceptibility of *P. larvae* strains infecting the larvae to FA.

An interesting finding in this study concerns the ERIC genotype characteristics of the Slovak *P. larvae* isolates. All 33 isolates collected over a large interval of 15 years had the ERIC II genotype. This result is surprising in the context of studies performed in several other European countries. These studies have shown that the ERIC II genotype occurs together with the ERIC I genotype in all these countries [10,58]. In Austria, a neighboring country to Slovakia, a prevalence of 58% for the ERIC I genotype among isolates has been reported [76]. This finding could reflect the specific evolution of *P. larvae* in Slovakia in the past and/or it could be associated with Slovak regulations that prohibited the import of foreign honeybee subspecies into the country and this could prevent the dissemination of *P. larvae* strains with ERIC I genotype in Slovakia. However, more data associated with our findings are needed in order to validate these explanations.

## 4. Materials and Methods

### 4.1. Fatty Acid

10-HDA of 98% purity was purchased from AK Scientific, Inc. (Union City, CA, USA). The crystalline powder of 10-HDA was stored at −20 °C. Methanolic stock solutions of 10-HDA were prepared fresh before each experiment.

### 4.2. P. larvae Strains

Six reference strains of *P. larvae* (CCUG 28515, LMG 9820, CCM 4483, CCM 4486, CCM 38 and CCM 39) were purchased from different culture collections. The 33 field isolates were obtained from infected material of AFB positive hives (ground stocks or decomposed larval samples) from apiaries located in different regions of Slovakia in 1999, 2009, 2011 and 2014 (Table 1). All isolates were verified to be *P. larvae* by the specific amplification of 16S rDNA using the specific primers PL1 and PL2 as described by Dobbelaere et al. [14].

### 4.3. ERIC-PCR Genotyping

The ERIC genotypes of *P. larvae* strains were determined by specific repetitive element PCR. The PCR reactions (20 μL) contained 2x HotStartTaq Master Mix (Qiagen, Venlo, The Netherlands), 1 μM primers ERIC1R 5′-ATGTAAGCTCCTGGGGATTCAC-3′ and ERIC2 5′-AAGTAAGTGACTGGGGTGAGCG-3′ [77] and 2 μL of DNA solution. The amplification conditions were the same as described Genersch et al. [10].

### 4.4. Cultivation of P. larvae

Vegetative cells were cultivated in two growth media, each prepared in several variants at different pH levels. One medium was MYPGP [78]; the other designated as MYPGP+P was a modified version of MYPGP containing 50 mM PIPES (Sigma-Aldrich, St. Louis, MI, USA) as a pH buffering compound. The media with different pH values were freshly prepared for each experiment. The bacterial cultivations were performed at 35 °C (the ordinary temperature in bee hives) as follows. Overnight cultures of the examined strains were prepared in standard MYPGP (pH = 7.2) on an orbital shaker and then diluted in media with different pH levels to a final concentration of 1 × 10^5^ cells/mL. Then, 150 μL aliquots of diluted bacterial culture were transferred into the wells of a sterile 96-well polystyrene microplates in triplicate and incubated stationarily for 43 h, whereby the plates were shaken on microplate shaker (Biofil, Merci, Paris, France) at 1200 rpm for 5 min before and after 18 h and 43 h of cultivation. Bacterial growth was determined spectrophotometrically by measuring the absorbance at 630 nm using a plate reader. All cultivations were repeated three times.

### 4.5. Preparation of Spores

*P. larvae* cells of selected strains (~1 000 CFU) were cultivated on MYPGP agar plates (pH = 7.2) at 35 °C for 10 days. Grown bacterial mass was removed from the surface of agar plates, homogenised and washed three times by vortexing and centrifugation at 15,000 *g* for 15 min in 15 mL of sterile deionised water in 15 mL centrifuge tubes (Labcon, Petaluma, CA, USA). Pelleted material was suspended in 2 mL of sterile deionised water and vegetative cells were killed by heating at 65 °C for 15 min. The spores were purified from the obtained suspensions by centrifugation on a Nycodenz (Axis-Shield PoC, Dundee, Scotland) gradient. Briefly, 2 mL of the suspensions were mixed with 11 mL of 30% sterile Nycodenz water solution in 15 mL centrifuge tubes. The mixtures were frozen twice at −20 °C and thawed at laboratory temperature to form gradients and then centrifuged at 15,000 *g* for 60 min. The content of the tubes was discarded and spores adhering to the tubes, visible as small pellets, were homogenised and washed three times with sterile deionised water as described above. Finally, 2 mL spore stock cultures were prepared in sterile deionised water and stored at 4 °C. The numbers of vital spores (CFU) in stock cultures were determined on standard MYPGP agar plates supplemented with 3 mM uric acid and L-tyrosine [79] after cultivation at 35 °C for 5 days.

### 4.6. Minimum Inhibitory Concentration Assay

MIC of 10-HDA was determined by the broth microdilution method using a modified MYPGP+P medium at variable pH. Cultures of bacterial vegetative cells or vital spores were prepared in the corresponding media at a concentration of 1 × 10^5^ cells or spores per 1 mL and aliquots of 147 μL were transferred into a sterile 96-well polystyrene microplate. A total of 3 μL from several 10-HDA stock solutions in methanol were added into the wells to prepare cultures with the concentrations of the substance in the range between 0.2 and 3 μg/μL with increments of 0.2 μg/μL. Positive and negative growth controls contained 3 μL of methanol. The microplates with *P. larvae* vegetative cells were incubated stationarily at 35 °C for 43 h, whereby they were shaken for 5 min on a rotary microplate shaker before and after 18 h and 43 h of cultivation. The spores were stationarily cultivated for 115 h and shaking was performed before and after 72, 96 and 115 h of cultivation. The bacterial growth inhibition was determined by monitoring the absorbance at 630 nm. The MIC was defined as the lowest concentration of 10-HDA inhibiting 100% of bacterial growth. All tests were performed in triplicate and were repeated three times. Finally, the mean of MIC values and standard deviations were determined.

## 5. Conclusions

This work expands our knowledge about substances contained in LJ that could inhibit the propagation of *P. larvae* in the midguts of individual larvae. Our results suggest that 10-HDA could play a significant role among these substances. We revealed that the inhibitory effects of 10-HDA against this pathogen are stronger at more acidic pH values thought to occur in the midguts of young larvae. Moreover, we found that environmentally important *P. larvae* strains (their vegetative form and also germinating spores) exhibited considerable differences in susceptibility to 10-HDA. We believe that these findings support our idea that larval food could play an important role in determining the resistance of individual larvae against *P. larvae*. We infer that variations in the contents of free molecules of 10-HDA and other active substances in LJ (not yet clearly identified) and in their inhibitory efficacies against different *P. larvae* strains as well as variations in the acidity and pH of LJ in larval midguts on the level of individual larvae within a colony and in different colonies can contribute to differences in colony resistance to AFB. However, further *in vivo* research is needed to verify these statements. Future *in vivo* experiments should evaluate the inhibitory effects of larval foods with different compositions possessing various combinations of the parameters important for anti-*P. larvae* activity in larval midguts. Finally, we believe that our data might be useful for the prospective breeding of new honeybee lines with improved larvae and colony resistance to AFB.

## Figures and Tables

**Table 1:** *P. larvae* strains, their characteristics and susceptibility to 10-HDA.

Strain	Source	ERIC Genotype	MIC* (μg/μL)
CCUG 28515	CCUG	I	1.05 ± 0.16
LMG 9820	LMG	I	0.60 ± 0.00
CCM 4483	CCM	I	1.20 ± 0.00
CCM 4486	CCM	II	1.28 ± 0.15
CCM 38a	CCM	III	2.80 ± 0.00
CCM 38b	CCM	IV	2.60 ± 0.00
CCM 39	CCM	III	2.60 ± 0.00
1-99	diseased brood	II	1.47 ± 0.14
2-99	diseased brood	II	1.40 ± 0.00
3-99	diseased brood	II	1.02 ± 0.18
4-99	diseased brood	II	1.27 ± 0.14
1-09	diseased brood	II	1.20 ± 0.00
3-09	diseased brood	II	1.78 ± 0.04
4-09	diseased brood	II	1.11 ± 0.09
5-09	diseased brood	II	1.40 ± 0.00
6-09	diseased brood	II	1.58 ± 0.04
7-09	diseased brood	II	1.35 ± 0.09
1-11	ground stock	II	0.98 ± 0.04
2-11	ground stock	II	1.35 ± 0.09
4-11	ground stock	II	1.60 ± 0.00
7-11	ground stock	II	1.33 ± 0.11
8-11	diseased brood	II	1.38 ± 0.04
11-11	ground stock	II	1.50 ± 0.11
13-11	ground stock	II	1.18 ± 0.04
14-11	ground stock	II	1.20 ± 0.00
17-11	ground stock	II	0.93 ± 0.11
21-11	ground stock	II	1.78 ± 0.04
22-11	ground stock	II	1.50 ± 0.11
23-11	ground stock	II	1.38 ± 0.04
24-11	ground stock	II	1.40 ± 0.00
25-11	ground stock	II	1.20 ± 0.00
26-11	ground stock	II	1.43 ± 0.17
34-11	ground stock	II	1.58 ± 0.04
1-14	diseased brood	II	1.38 ± 0.04
2-14	diseased brood	II	1.58 ± 0.04
3-14	diseased brood	II	1.40 ± 0.00
4-14	diseased brood	II	0.60 ± 0.00
5-14	diseased brood	II	1.38 ± 0.04
6-14	diseased brood	II	1.00 ± 0.00
7-14	diseased brood	II	1.40 ± 0.00

* Determined at pH = 6.6.

**Table 2:** Antibacterial efficacy of 10-HDA on vegetative cells and spores of *P. larvae*.

Strain	ERIC Genotype	MIC (μg/μL) *	
		Vegetative Cells	Spores
CCUG 28515	I	1.05 ± 0.16	1.05 ± 0.16
LMG 9820	I	0.60 ± 0.00	0.60 ± 0.00
CCM 4483	I	1.20 ± 0.00	0.98 ± 0.04
CCM 4486	II	1.28 ± 0.15	0.60 ± 0.00
PL 2-11	II	1.35 ± 0.09	1.18 ± 0.04
PL 4-11	II	1.60 ± 0.00	1.20 ± 0.00

* MIC determined at pH = 6.6.

## Supplementary Materials

The following are available online at http://www.mdpi.com/1420-3049/23/12/3236/s1, Figure S1: ERIC-PCR genotyping of selected *P. larvae* strains.
