# Supplementary material for: 10-HDA, A Major Fatty Acid of Royal Jelly, Exhibits pH Dependent Growth-Inhibitory Activity Against Different Strains of Paenibacillus larvae
Source: Molecules. 2018 Dec 7;23(12):3236. doi: 10.3390/molecules23123236 (PMC6320966; doi:10.3390/molecules23123236)
Supplement: Supplementary file 1 [file molecules-23-03236-s001.pdf]

Supplementary materials

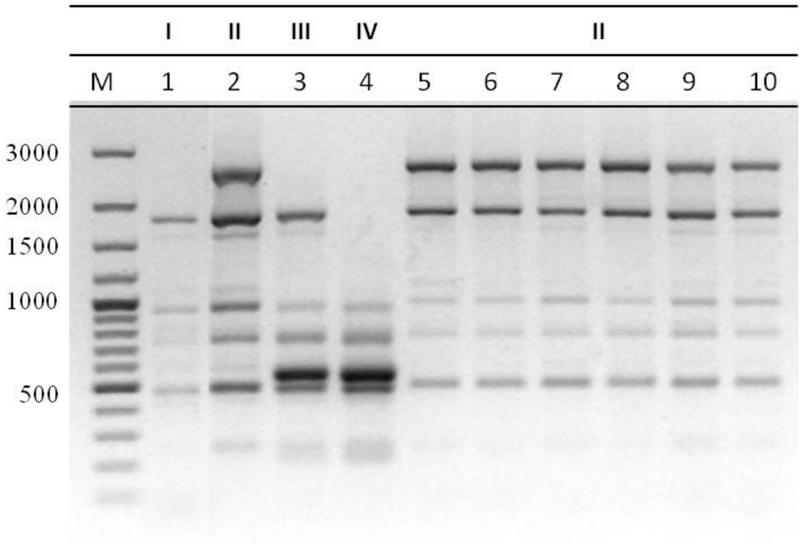

Figure S1. ERIC-PCR genotyping of selected *P. larvae* strains. Lane M - 100 bp marker; lanes 1-4 - ERIC I-ERIC IV electrophoretic patterns of reference strains CCUG 28515, CCM 4486, CCM 39 and CCM 38b, respectively; lanes 5-10 - ERIC II patterns of several field isolates. PCR products were analysed on a 1% agarose gel containing GelRed stain.
